# Supplementary material for: The Effect of Supplementation with Betaine and Zinc on In Vitro Large Intestinal Fermentation in Iberian Pigs under Heat Stress
Source: Animals (Basel). 2023 Mar 20;13(6):1102. doi: 10.3390/ani13061102 (PMC10044697; doi:10.3390/ani13061102)
Supplement: Supplementary file 1 [file animals-13-01102-s001.zip › animals-2201951-supplementary.pdf]

Table S1. Chemical composition of the control diet (g/kg as fed).

**Ingredients**	
Barley grain	700
Corn	144
Soybean meal	127
Calcium phosphate	9.3
Calcium carbonate	6.2
Sodium chloride	3.0
L-Lysine (50%)	5.0
L-Threonine (50%)	2.1
Methionine hydroxy-analog (75%)	0.7
Vitamins and minerals[1]	3.0
**Chemical analysis**	
Dry matter	899
Ash	48.6
Ether extract	17.5
Crude protein	146
Gross energy (MJ/kg)	16.6
aNDFom[2]	141
ADFom[3]	44.5
Lignin(sa)[4]	2.1

[1] Provided (per kg of diet): 2000 UI retinol as retinyl acetate, 800 UI cholecalciferol, 40 UI dL-α-tocopheryl acetate, 1.5 mg menadione, 2 mg thiamine, 3 mg riboflavin, 50 µg cyanocobalamin, 15 µg folic acid, 22.5 mg nicotinic acid, 15 mg d-pantothenic acid, 60 mg MnO, 80 mg $FeCO_3$, 80 mg ZnO, 750 µg KI, 10 mg $CuSO_4 \times 5H_2O$, 50 µg Na_2SeO_3, 250 mg sepiolite, 1.5 mg butylhydroxyanisole (BHA), and 7.5 mg butylhydroxytoluene (BHT). [2] aNDFom, neutral detergent fiber inclusive of ash residual. [3] ADFom, acid detergent fiber exclusive of residual ash. [4] Lignin(sa), lignin determined by solubilization of cellulose with sulfuric acid.

Table S2. Target bacterial groups quantified, primer sequences, amplicon sizes, type strains used as standards, and conditions of culture of the type strains.

**Target**	**F primer**	**R primer**	**Amplicon size**	**Type strain**	**Culture conditions**	**Final concentration**
Total bacteria	CGG<br>TGA<br>ATA<br>CGT<br>TCC<br>CGG	TAC<br>GGC<br>TAC<br>CTT<br>GTT<br>ACG<br>ACT T	142	Escherichia coli ATCC 11775	24 h, anaerobiosis	1011 cfu/mL
Enterobacteriaceae	ATG<br>GCT<br>GTC<br>GTC<br>AGC<br>TCG T	CTA<br>CTT<br>CTT<br>TTG<br>CAA<br>CCC<br>ACT C	385	Escherichia coli ATCC 11775	24 h, anaerobiosis	1011 cfu/mL

Lactobacillus	AGC AGT AGG GAA TCT TCC A	CAC CGC TAC ACA TGG AG	337-341	Lactobacillus plantarum CECT 748	24 h, anaerobiosis	1010 cfu/mL
Bifidobacterium	CGC GTC YGG TGT GAA AG	GGT GTT CTT CCC GAT ATC TAC A	127	Bifidobacterium bifidum CECT 870	48 h, anaerobiosis	1010 cfu/mL
Bacteroides	GAG AGG AAG GTC CCC CAC	CGC TAC TTG GCT GGT TCA G	106	Bacteroides coprosuis DSM 18011	48 h, anaerobiosis	1010 cfu/mL
Clostridium cluster IV	ACA GGT GGT GCA TGG TTG TC	AGA GTG CTC TTG CGT AG	101	Clostridium leptum DSM 753	48 h, anaerobiosis	109 cfu/mL
Clostridium cluster XIV	AAA TGA CGG TAC CTG ACT AA	CTA CGC WCC CTT TAC AC	102	Blautia coccoides DSM 935	48 h, anaerobiosis	109 cfu/mL
